# Bacterial fecal microbiota is only minimally affected by a standardized weight loss plan in obese cats

**DOI:** 10.1186/s12917-020-02318-2

**Published:** 2020-04-15

**Authors:** Moran Tal, J. Scott Weese, Diego E. Gomez, Myriam Hesta, Joerg M. Steiner, Adronie Verbrugghe

**Affiliations:** 1 Department of Clinical Studies, Ontario Veterinary College, University of Guelph, Guelph, ON N1G 2W1 Canada; 2 Present address: Royal Canin Canada, 100 Beiber Rd, N0B 2J0 Puslinch, Canada; 3 Department of Pathobiology, Ontario Veterinary College, University of Guelph, Guelph, ON N1G 2W1 Canada; 4 Laboratory of Animal Nutrition, Faculty of Veterinary Medicine, Ghent University, Merelbeke, B-9820 Belgium; 5 Gastrointestinal Laboratory, Department of Small Animal Clinical Sciences, College of Veterinary Medicine and Biomedical Sciences, Texas A&M University, College Station, 77843 TX USA

**Keywords:** Feline obesity, Fecal microbiome weight loss, Microbial diversity, Folate, Cobalamin

## Abstract

**Background:**

Research in humans and mice suggests that obesity influences the abundance and diversity of gastrointestinal (GI) microbiota, and that an “obese microbiome” influences energy metabolism and fat storage in the host. Microbiota membership and composition have been previously assessed in healthy cats. However, research investigating the effects of obesity and weight loss on the cat’s fecal microbiota is limited. Therefore, this study’s objective was to evaluate differences in fecal microbial abundance and biodiversity, as well as serum cobalamin and folate concentrations in obese cats, before and after weight loss, and compare to lean cats.

Fourteen lean and 17 obese healthy client-owned cats were fed a veterinary therapeutic weight loss food at maintenance energy requirement for 4 weeks. At the end of week 4, lean cats finished the study, whereas obese cats continued with a 10-week weight loss period on the same food, fed at individually-tailored weight loss energy requirements. Body weight and body condition score were recorded every 2 weeks throughout the study. At the end of each period, a fecal sample and food-consumption records were obtained from the owners, and serum cobalamin and folate concentrations were analysed. DNA was extracted from fecal samples, polymerase chain reaction (PCR) was performed, and products were sequenced using next-generation sequencing (Illumina MiSeq).

**Results:**

No significant differences in the relative abundance of taxa and in biodiversity indices were observed between cats in either group (*P* > 0.05 for all tests). Nevertheless, some significantly enriched taxa, mainly belonging to Firmicutes, were noted in linear discriminant analysis effect size test in obese cats before weight loss compared to lean cats. Serum cobalamin concentrations were significantly higher in lean compared to obese cats both before and after weight loss. Serum folate concentrations were higher in obese cats before weight loss compared to after.

**Conclusions:**

The association between feline obesity and the fecal bacterial microbiota was demonstrated in enriched taxa in obese cats compared to lean cats, which may be related to enhanced efficiency of energy-harvesting. However, in obese cats, the fecal microbial abundance and biodiversity were only minimally affected during the early phase of a standardized weight loss plan.

## Background

Obesity is a prominent problem in both humans and companion animals. There are a few definitions for obesity, but they are all in agreement that the term reflects an excess of body fat that jeopardizes the individual’s health [1–4]. Estimations from the last decade indicate that in Europe, 11.5 and 26.8% of cats are overweight or obese in the UK and France respectively, while 59.5% of cats were reported as overweight of obese in the USA and 63% in New Zealand [5–8].

Obesity is mainly caused by an imbalance between energy intake and energy expenditure [9]. Additional factors contribute to obesity, such as genetics, age, sex, neutering, physical inactivity, and endocrine disease [7, 8, 10–12]. However, more recent findings indicate that the gastrointestinal (GI) microbiota (i.e., the gastrointestinal microbial community) plays an important role in the development of obesity. The microbiota can be addressed as a “metabolic organ”, acting to support the host with metabolic functions that the host is not capable of performing, such as fermentation of plant polysaccharides [13–15]. The microbiota can also affect metabolic pathways in the host, for example promote metabolic pathways that enhance adipose tissue storage [13, 16]. Moreover, the microbiota is also involved in low-grade inflammation, which occurs with obesity. It is suggested that lipopolysaccharide (LPS), a structural component of the external membrane of gram-negative bacteria, triggers inflammation in the host through the innate immune response [17].

Increased relative abundance of Firmicutes versus Bacteroidetes, and hence a higher Firmicutes-to-Bacteroidetes ratio were observed in obese humans and mice compared to their lean counterparts [18, 19]. However, some studies did not observe these differences [15]. Obesity was also associated with reduced microbial richness and diversity in humans and mice [20, 21]. Nonetheless, little research exists in companion animals to demonstrate similar findings. Research in dogs demonstrated increased relative abundance of the phylum Actinobacteria and genus *Roseburia* in lean versus obese dogs [22]. However, the subjects differed in their environment (i.e., lean research dogs and obese pet dogs), and the dogs were not fed the same food [22]. Limited research on the relationship between obesity and the fecal microbiota has also been performed in cats. One study examined the differences of the microbiota between lean and overweight/obese cats using two fecal collection methods – rectal swabs versus litter box samples. Microbiota differences were found between lean and obese cats with both collection methods. However, shelter cats were used, with the medical history being unknown, and various different diets being consumed (i.e., no diet adaptation period). Hence, the results may have been affected by the diet or an unknown medical condition. Also the effect of weight loss was not assessed [23]. A second feline study examined the effect of weight loss on the microbiota in eight obese kennel cats. However, the sample size was quite small, results were not compared to matching lean kennel cats, and the study environment was well controlled, and thus, less reflective of the true effects in the general cat population [24]. A third feline study, examined the effects of body composition, 6 weeks of energy restriction and neutering on the feline fecal mictobiota of cats. Nonetheless, the weight loss period was quite short, the sample size was small and the cats were specific-pathogen-free kennel cats, so again not reflective of the general pet cat population [25].

Obesity may impact intestinal permeability through deviation from normal microbial structure (i.e., bacterial dysbiosis), which could cause an increase in lipopolysaccharide (LPS)-producing bacteria and overall LPS production [26]. The direct assessment of intestinal permeability via tight junction protein expression, for example, requires harvesting of intestinal tissue [27]. However, measuring serum cobalamin and folate can be used for non-invasive assessment of intestinal health in humans, dogs, and cats [28, 29]. Gastrointestinal bacterial dysbiosis can be a cause of reduced serum concentrations of cobalamin and may alter microbial folate synthesis [28]. Low serum cobalamin and/or folate status have been observed in various obese human populations, for example children and adolescents, post-menopausal women and pre-operative bariatric surgery patients. It is however uncertain if this is due to volumetric dilution of the blood of obese patients, low dietary intake, reduced uptake by the intestinal epithelium, increased catabolism, and sequestration in adipose tissue, or changes in the gut microbiota profiles [30–33]. To the authors’ knowledge, serum cobalamin and folate concentrations have not been assessed in feline obesity.

Therefore, the aim of the current study was to investigate the fecal microbiota and serum cobalamin and folate concentrations in obese client-owned cats before and after a 10-week standardized weight loss plan and to compare these to a lean control group, while all cats were acclimatized for 4 weeks to the same diet before study enrollment. It was hypothesized that the feline fecal microbial composition and structure will differ between lean and obese cats and that these microbial differences will revert with weight loss in obese cats, to resemble the fecal microbiota of lean cats. It was also hypothesized that a reduced cobalamin concentration and increased folate concentration will be observed in obese cats before weight loss compared with lean cats and obese cats after weight loss.

## Results

Fourteen lean and 17 obese cats were enrolled into the study. One obese cat did not complete the study due to reduced owner cooperation with food restriction, which resulted in weight maintenance rather than weight loss. Results from this cat were included for the obese cats before weight loss (OBWL) time point, but not for the obese cats after weight loss (OAWL) time point. Only one stool sample and food intake data were obtained for one cat because of its fractious nature, leaving 13 cats for assessment of body weight (BW) and body composition in the LEAN group. All cats tolerated the food well, no cat refused to eat the food, and none showed signs of illness or maldigestion (mean food consumption ± standard deviation (SD) within groups – LEAN: 215.2 ± 35.69 kcal, OBWL: 217.8 ± 24.33 kcal, OAWL: 134.6 ± 9.20 kcal).

### Body weight and body composition measurements

Body weight remained stable in all cats during the 4-week adaptation period. However, mean BW was significantly lower in the LEAN versus the OBWL (*P* < 0.0001) group. The 10-week weight loss plan was successful as body weight was significantly lower for OAWL compared to OBWL (*P* < 0.0001) measurements. On average cats lost 0.94 ± 0.28% of their initial body weight per week. Also, a significant decrease in body condition score (BCS) between OBWL and OAWL was observed (*P* = 0.001). Body mass index (BMI) and girth in LEAN were significantly lower compared to OBWL (*P* < 0.0001), and were also significantly lower for OAWL compared to OBWL (*P* < 0.0001). Altogether, BW, BCS, BMI and girth were still significantly higher in the obese cat group than in LEAN cats after the 10-week weight loss plan (*P* < 0.0001 for BW, BCS and girth, and *P* < 0.0002 for BMI) (Table 1).

**Table 1:** Body weight and body composition measurements in lean and obese cats before and after weight loss

	LEAN	OBWL	OAWL
	Mean ± SD	Mean ± SD	Mean ± SD
BW (kg)	4.49 ± 0.22^a,c^	6.95 ± 1.32^a,b^	6.30 ± 1.13^b,c^
BMI (kg/m^2^)	41.58 ± 4.67^a,c^	60.45 ± 12.05^a,b^	55.61 ± 11.02^b,c^
Girth (cm)	38.38 ± 3.79^a,c^	52.12 ± 4.89^a,b^	48.25 ± 5.55^b,c^
	Median (Min-Max)	Median (Min-Max)	Median (Min-Max)
BCS (1–9/9)	5 (4 to 5)^a,c^	9 (8 to 9)^a,b^	8 (6 to 9)^b,c^

*OBWL* obese before weight loss, *OAWL* obese after weight loss, *BW* body weight, *BMI* body mass index, *BCS* body condition score, *SD* standard deviation, *Min* minimum, *Max* maximum

The data presented here represent the BW and body composition measurements of healthy lean cats (LEAN, *n* = 13) and obese cats following a 4-week adaptation period with a veterinary therapeutic food intended for weight loss and adult maintenance (OBWL, *n* = 17), and obese cats after a 10-week weight loss period on the same food (OAWL, *n* = 16)

^a^Significant difference between LEAN to OBWL (*P* < 0.0001for BW, girth, BMI and BCS); Student T-test (BW, BMI and girth) or Wilcoxon Mann-Whitney (BCS)

^b^Significant difference between OBWL to OAWL (*P* < 0.0001 for BW, girth and BMI; *P* = 0.001 for BCS); Paired T-test (BW, BMI and girth) or Wilcoxon Signed-Rank (BCS)

^c^Significant difference between LEAN to OAWL (*P* < 0.0001 for BW, girth, and BCS; *P* < 0.0002 for BMI); Student T-test (BW, BMI and girth) or Wilcoxon Mann-Whitney (BCS)

### Serum Cobalamin and Folate concentrations

Serum cobalamin concentrations were higher in LEAN cats compared to OBWL cats (*P* = 0.009) and were also higher in the LEAN group compared to the OAWL group (*P* = 0.021). No differences were observed in serum concentrations of cobalamin between OBWL and OAWL measurements. Serum folate concentrations were significantly higher for OBWL compared to OAWL (*P* = 0.003). No other differences were observed for serum folate concentrations between groups or time points (Table 2).

**Table 2:** Serum cobalamin and folate concentrations in lean cats and in obese cats before and after weight loss

Analytes	LEAN	OBWL	OAWL
	Median (Min-Max)	Median (Min-Max)	Median (Min-Max)
Cobalamin (pg/mL) (350–1499 pg/mL)^a^	913 (821–973) ^b,d^	882.0 (702–928) ^b^	879.0 (571–974) ^d^
	Mean (LL-UL)	Mean (LL-UL)	Mean (LL-UL)
Folate (ng/mL) (9.7–21.6 ng/mL)^a^	18.8 (16.2–21.8)	20.1 (17.7–22.9) ^c^	17.4 (15.3–19.9) ^c^

*OBWL* obese before weight loss, *OAWL*, obese after weight loss, *Min* minimum, *Max* maximum, *LL-UL* lower limit – upper limit

The data presented here represent the cobalamin and folate serum concentrations of healthy lean cats (LEAN, *n* = 13) and obese cats following a 4-week adaptation period with a veterinary therapeutic food intended for weight loss and adult maintenance (OBWL, *n* = 17), and obese cats after a 10-week weight loss period on the same food (OAWL, *n* = 16)

^a^Normal reference-range provided by the Gastrointestinal Laboratory, Texas A&M University

^b^Significant difference between LEAN to OBWL (and *P* = 0.0057 for cobalamin and *P* = 0.2989 for folate); Wilcoxon Mann-Whitney (cobalamin) or Student T-test after log transformation (folate)

^c^Significant difference between OBWL to OAWL (*P* = 0.8209 for cobalamin and *P* = 0.0321 for folate); Wilcoxon Signed Rank (cobalamin) or Paired T-test after log transformation (folate)

^d^Significant difference between LEAN to OAWL (*P* = 0.0149 for cobalamin and *P* = 0.6058 for folate); Wilcoxon Mann-Whitney (cobalamin) or Student T-test after log transformation (folate)

### Fecal microbiota analyses

Fecal analyses resulted in a total of 9,193,399 sequences that passed all filters, with a median of 105,984 sequences per sample (range: 22,314-557,519). A random subsample of 22,314 sequences per sample was used for sample normalization. One thousand forty-two operational taxonomic units (OTUs) were formed.

#### Relative abundance

**Fig. 1:**
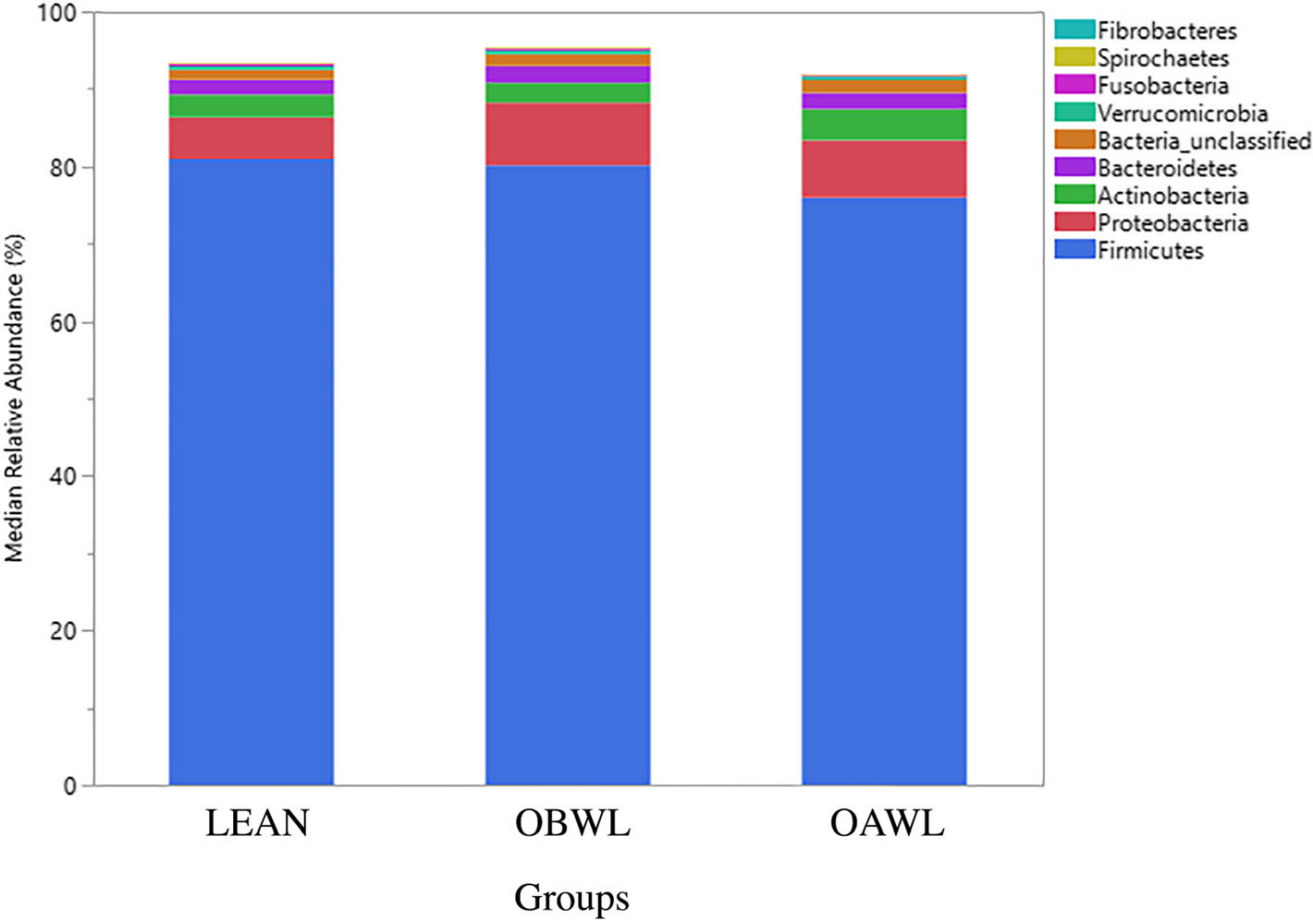
The relative abundances of predominant phyla originating from fecal samples of healthy lean cats (LEAN, *n* = 14) and obese cats (OBWL, *n* = 17) following a 4-week adaptation period with a veterinary therapeutic food intended for weight loss and adult maintenance, and obese cats after a 10-week weight loss period on the same food (OAWL, *n* = 16)

**Fig. 2:**
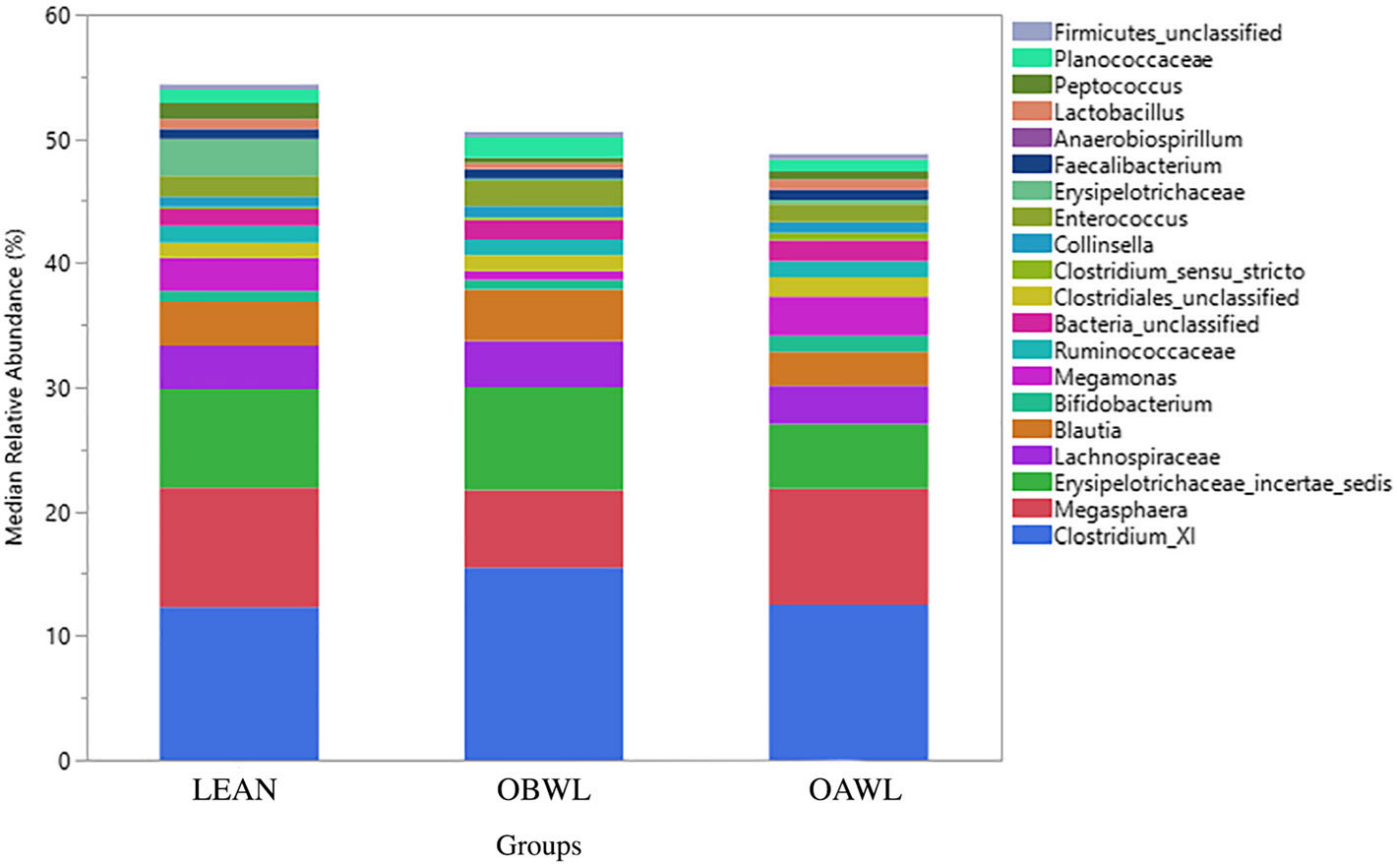
The relative abundances of predominant genera originating from fecal samples of healthy lean cats (LEAN, *n* = 14) and obese cats (OBWL, *n* = 17) following a 4-week adaptation period with a veterinary therapeutic food intended for weight loss and adult maintenance, and obese cats after a 10-week weight loss period on the same food (OAWL, *n* = 16)

Median relative bacterial abundance was examined across all taxa between groups. After performing a Benjamini-Hochberg adjustment, significant differences between groups were no longer identified for any taxa (Figs. 1 & 2, Table 3, *P* > 0.05). The most abundant phyla in all groups were (in a descending order) Firmicutes, Proteobacteria, Actinobacteria, and Bacteroidetes. The most abundant genera in all groups were (in a descending order): Clostridium_XI, Megasphaera, Erysipelotrichaceae_incertae_sedis, Lachnospiraceae and blautia.

**Table 3:** Relative abundances of the fecal microbiota members of healthy lean cats and obese cats before and after weight loss

	Medians (Min-Max) of relative abundance (%)		
	LEAN	OBWL	OAWL
Phyla (9/9)^a^			
Firmicutes	81.0 (63.7–91.0)	80.2 (5.3–93.4)	76.0 (46.3–92.1)
Proteobacteria	5.4 (1.9–23.2)	8.2 (2.9–19.0)	7.4 (1.2–43.0)
Actinobacteria	2.9 (0.8–32.8)	2.5 (0.4–30.1)	4.0 (0.5–13.6)
Bacteroidetes	1.9 (0.4–5.1)	2.1 (0.5–4.3)	2.0 (0.6–5.3)
Bacteria_unclassified	1.3 (0.5–10.4)	1.6 (0.3–3.7)	0.7 (0.0–3.4)
Verrucomicrobia	0.4 (0.1–7.3)	0.5 (0.1–2.5)	0.4 (0.0–7.8)
Fusobacteria	0.3 (0.0–1.5)	0.3 (0.0–5.7)	0.2 (0.0–3.5)
Spirochaetes	0.1 (0.0–3.7)	0.1 (0.0–1.2)	0.1 (0.0–2.13)
Fibrobacteres	0.0 (0.0–1.1)	0.0 (0.0–0.7)	0.0 (0.0–0.8)
Class (10/25)^a^			
Clostridia	35.8 (13.1–59.3)	41.1 (20.1–70.9)	38.6 (17.8–68.0)
Negativicutes	15.4 (5.1–36.1)	9.0 (0.1–43.3)	14.6 (1.3–39.1)
Eryspelotrichia	16.8 (0.72–33.3)	10.6 (0.3–25.3)	7.7 (0.4–15.0)
Bacilli	8.2 (2.4–33.3)	9.8 (2.1–32.0)	6.2 (0.6–29.8)
Actinobacteria	2.9 (0.8–32.8)	2.5 (0.4–30.1)	4.0 (0.5–13.6)
Gammaproteobacteria	2.9 (0.8–17.0)	4.7 (1.0–16.0)	4.4 (0.1–35.8)
Bacteria_unclassified	1.3 (0.5–10.4)	1.6 (0.3–3.7)	1.7 (0.0–6.4)
Bacteroidia	1.5 (0.4–4.7)	1.6 (0.4–3.6)	1.5 (0.3–5.1)
Firmicutes_unclassified	0.4 (0.1–4.2)	0.4 (0.1–1.8)	0.5 (0.0–5.3)
Alphaproteobacteria	0.9 (0.4–4.0)	1.0 (0.4–3.4)	0.8 (0.0–3.6)
Order (15/56)^a^			
Clostridiales	35.6 (12.9–59.3)	40.7 (20.1–70.9)	38.2 (17.7–68.0)
Selenomonadales	15.4 (5.1–36.1)	9.0 (0.1–43.3)	14.6 (1.3–39.1)
Erysipelotrichales	16.8 (0.7–33.3)	10.6 (0.3–25.3)	7.7 (0.4–15.0)
Lactobacillales	4.7 (1.2–15.3)	5.8 (1.2–14.8)	5.2 (0.3–14.3)
Bacillales	3.0 (0.7–17.9)	3.7 (0.9–17.1)	2.2 (0.2–17.3)
Bifidobacteriales	0.9 (0.4–29.0)	0.7 (0.1–24.9)	1.3 (0.2–11.9)
Coriobacteriales	1.1 (0.3–4.8)	1.3 (0.2–5.1)	1.4 (0.2–7.4)
Bacteria_unclassified	1.3 (0.5–10.4)	1.6 (0.3–3.7)	1.7 (0.0–6.4)
Bacteroidales	1.5 (0.38–4.7)	1.6 (0.4–3.6)	1.5 (0.3–5.1)
Aeromonadales	0.2 (0.0–9.4)	0.9 (0.0–6.9)	0.6 (0.0–32.0)
Enterobacteriales	0.5 (0.1–1.6)	0.7 (0.1–10.6)	0.7 (0.0–4.5)
Firmicutes_unclassified	0.4 (0.1–4.2)	0.4 (0.1–1.8)	0.5 (0.0–5.3)
Verrucomicrobiales	0.2 (0.1–1.6)	0.3 (0.1–0.9)	0.3 (0.0–7.5)
Campylobacterales	0.4 (0.0–10.5)	0.3 (0.0–3.1)	0.4 (0.0–3.6)
Xanthomonadales	0.3 (0.1–1.0)	0.4 (0.1–1.0)	0.4 (0.0–7.1)
Family (20/99)^a^			
Peptostreptococcaceae	13.1 (4.1–32.5)	16.5 (0.6–53.0)	12.8 (0.3–50.7)
Veilloncellaceae	14.2 (4.5–36.1)	8.8 (0.1–40.8)	14.3 (0.4–39.1)
Erysipelotrichaceae	16.8 (0.7–33.3)	10.6 (0.3–25.3)	7.7 (0.4–15.0)
Lachnospiraceae	9.7 (2.7–23.1)	9.7 (6.2–23.2)	8.4 (3.8–17.0)
Ruminococcaceae	3.6 (1.2–13.1)	3.4 (1.2–9.7)	5.9 (0.7–11.6)
Bifidobacteriaceae	0.9 (0.4–29.0)	0.7 (0.1–24.9)	1.3 (0.2–11.9)
Clostridiales_unclassified	1.2 (0.4–3.4)	1.3 (0.2–4.4)	1.5 (0.5–11.1)
Clostridiaceae_1	0.3 (0.1–5.7)	0.6 (0.1–16.6)	1.4 (0.1–18.6)
Coriobacteriaceae	1.1 (0.3–4.8)	1.3 (0.2–5.1)	1.4 (0.2–7.4)
Enterococcaceae	1.9 (0.2–12.0)	2.3 (0.3–11.6)	1.5 (0.1–9.4)
Succinivibrionaceae	0.2 (0.0–9.3)	0.9 (0.0–6.8)	0.4 (0.0–32.0)
Planococcaceae	1.4 (0.3–8.0)	2.2 (0.6–8.3)	1.2 (0.1–8.3)
Lactobacillaceae	0.8 (0.0–6.3)	0.5 (0.1–11.2)	0.8 (0.1–4.6)
Enterobacteriaceae	0.5 (0.1–4.6)	0.7 (0.1–10.6)	0.7 (0.0–4.5)
Petptococcaceae_1	1.3 (0.0–3.5)	0.4 (0.0–5.3)	0.7 (0.0–4.1)
Firmicutes_unclassified	0.4 (0.1–4.2)	0.4 (0.1–1.8)	0.5 (0.0–5.3)
Bacteroidaceae	0.9 (0.2–2.7)	0.7 (0.2–3.1)	1.0 (0.1–2.8)
Acidaminococcaceae	0.7 (0.1–2.3)	0.3 (0.0–2.5)	0.2 (0.0–3.8)
Verrucomicrobiaceae	0.2 (0.1–1.6)	0.3 (0.0–7.5)	0.3 (0.1–0.9)
Genera (20/199)^a^			
Clostridium_XI	12.3 (3.9–32.4)	15.5 (0.5–51.3)	12.5 (0.3–50.6)
Megasphaera	9.6 (0.0–35.4)	6.2 (0.0–32.6)	9.3 (0.0–38.8)
Erysipelotrichaceae_incertae_sedis	8.0 (0.4–33.0)	8.3 (0.1–18.4)	5.2 (0.2–12.7)
Lachnospiraceae	3.5 (1.1–7.0)	3.7 (1.9–7.6)	3.0 (1.6–8.6)
Blautia	3.5 (0.9–13.0)	4.1 (1.4–7.7)	2.7 (0.6–8.5)
Bifidobacterium	0.9 (0.4–29.0)	0.7 (0.1–24.9)	1.3 (0.2–11.9)
Megamonas	2.6 (0.0–11.7)	0.8 (0.0–15.4)	3.1 (0.0–18.4)
Clostridiales_unclassified	1.22 (0.4–9.4)	1.3 (0.2–4.4)	1.5 (0.5–11.1)
Ruminococcaceae	1.4 (0.4–6.8)	1.2 (0.3–3.8)	1.3 (0.4–5.5)
Bacteria_unclassified	1.3 (0.5–10.4)	1.6 (0.3–3.7)	1.7 (0.0–6.4)
Clostridium_sensu_stricto	0.2 (0.0–1.4)	0.2 (0.1–15.9)	0.6 (0.1–18.3)
Collinsella	0.8 (0.2–3.4)	0.9 (0.2–2.2)	0.9 (0.1–6.7)
Enterococcus	1.6 (0.2–10.7)	2.0 (0.3–10.4)	1.4 (0.1–8.4)
Erysipelotrichaceae	3.0 (0.0–6.7)	0.2 (0.0–4.9)	0.3 (0.0–5.0)
Faecalibacterium	0.8 (0.0–2.4)	0.7 (0.1–3.6)	0.8 (0.0–7.0)
Anaerobiospirillum	0.0 (0.0–8.5)	0.0 (0.0–6.8)	0.1 (0.0–31.9)
Lactobacillus	0.7 (0.0–6.1)	0.5 (0.1–10.6)	0.7 (0.1–4.4)
Planococcaceae	1.3 (0.0–3.5)	0.4 (0.0–5.3)	0.7 (0.0–4.1)
Firmicutes_unclassified	0.4 (0.1–4.2)	0.4 (0.1–1)	0.5 (0.0–5.3)
Lachnospiraceae_incertae_sedis	0.9 (0.3–2.6)	1.1 (0.2–2.7)	1.0 (0.1–2.5)

*LEAN* lean cats, *OBWL* obese cats before weight loss, *OAWL* obese cats after weight loss

The relative abundances across taxa presented here represent the fecal microbiota of healthy lean cats (LEAN, *n* = 14) and obese cats (OBWL, *n* = 17) following a 4-week adaptation period with a veterinary therapeutic food intended for weight loss and adult maintenance, and obese cats after a 10-week weight loss period on the same food (OAWL, *n* = 16). Cut-off for phyla and genera in the study were 1 and 0.1%, respectively. However, only a portion of the most abundant members for all taxa (besides to phyla) are presented in the table. No significant differences were found between groups, using Wilcoxon Rank Sum and Wilcoxon Signed-Rank, depending on the groups’ comparison, followed by the Benjamini-Hochberg adjustment (*P* > 0.05 for all comparisons; not shown in the table)

^a^ The numbers after each taxa represent the number of members in the specific taxa presented in the table out of the overall number of members in that taxa retrieved by the analyses

#### Alpha and beta diversity indices

**Fig. 3:**
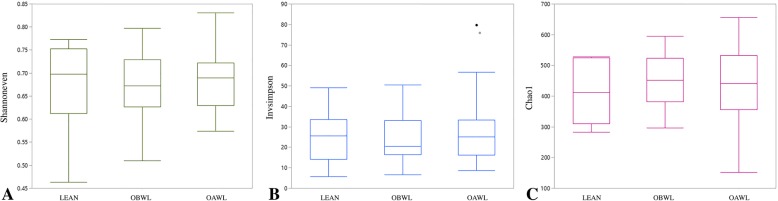
Bacterial population evenness (Shannoneven), diversity (Invsimpson), and richness (Chao1) in healthy lean cats (LEAN, *n* = 14) and obese cats (OBWL, *n* = 17) following a 4-week adaptation period with a veterinary therapeutic food intended for weight loss and adult maintenance, and obese cats after a 10-week weight loss period on the same food (OAWL, *n* = 16)

There were no significant differences in alpha diversity matrices between groups or time-points (all *P* > 0.05) (Fig. 3). There were also no differences in community membership (Classical Jaccard index: unifrac *P* = 0.17; parsimony *P* > 0.05 for all comparisons) or population structure (Yue & Clayton: unifrac *P* = 0.93; parsimony *P* > 0.05 for all comparisons) between groups or time-points.

**Fig. 4:**
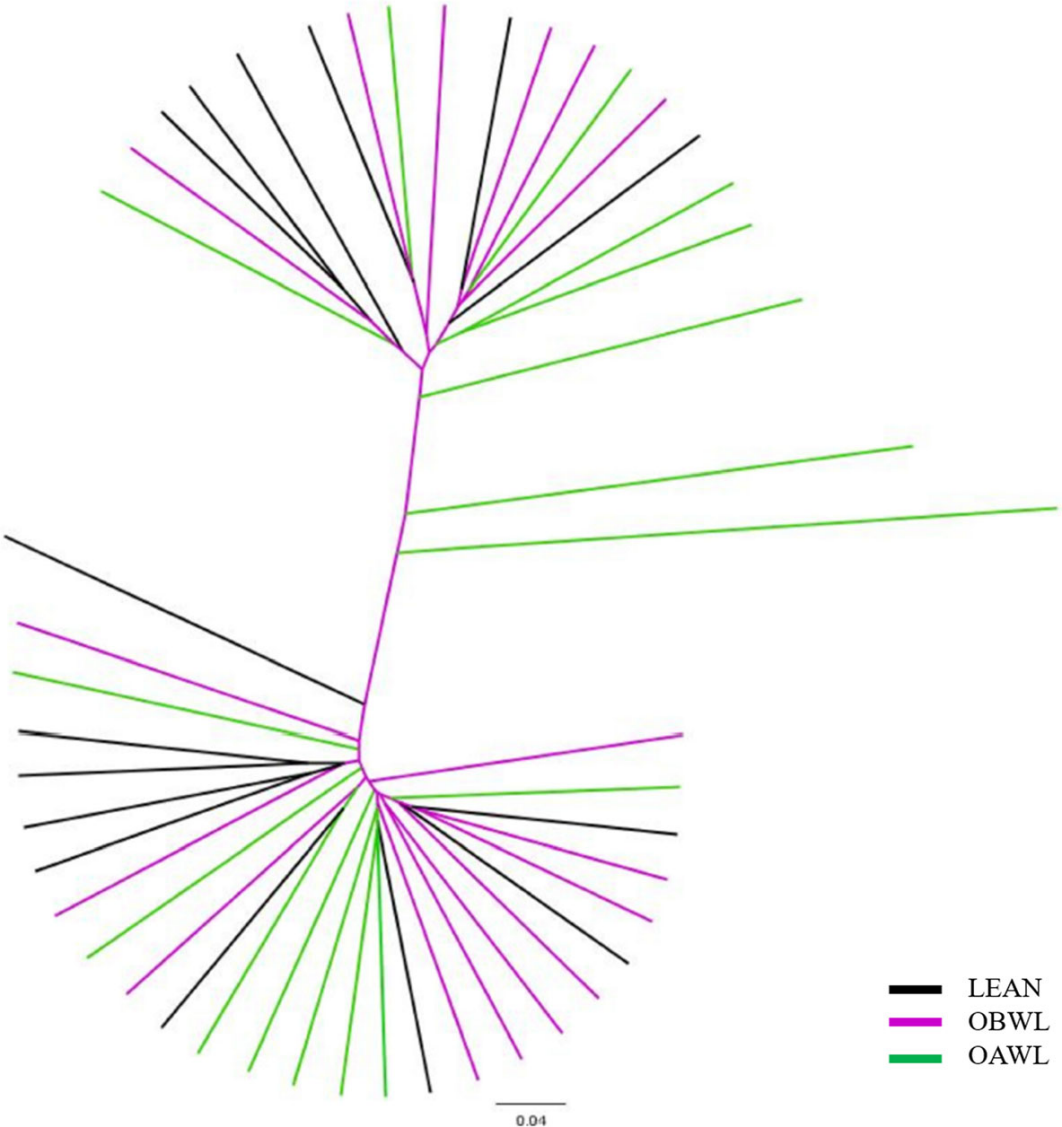
Dendogram of the Classical Jaccard index representing the community membership of the fecal microbiota in healthy lean cats (LEAN, *n* = 14) and obese cats (OBWL, *n* = 17) following a 4-week adaptation period with a veterinary therapeutic food intended for weight loss and adult maintenance, and obese cats after a 10-week weight loss period on the same food (OAWL, *n* = 16). Each group is represented with a different colour (see legend)

**Fig. 5:**
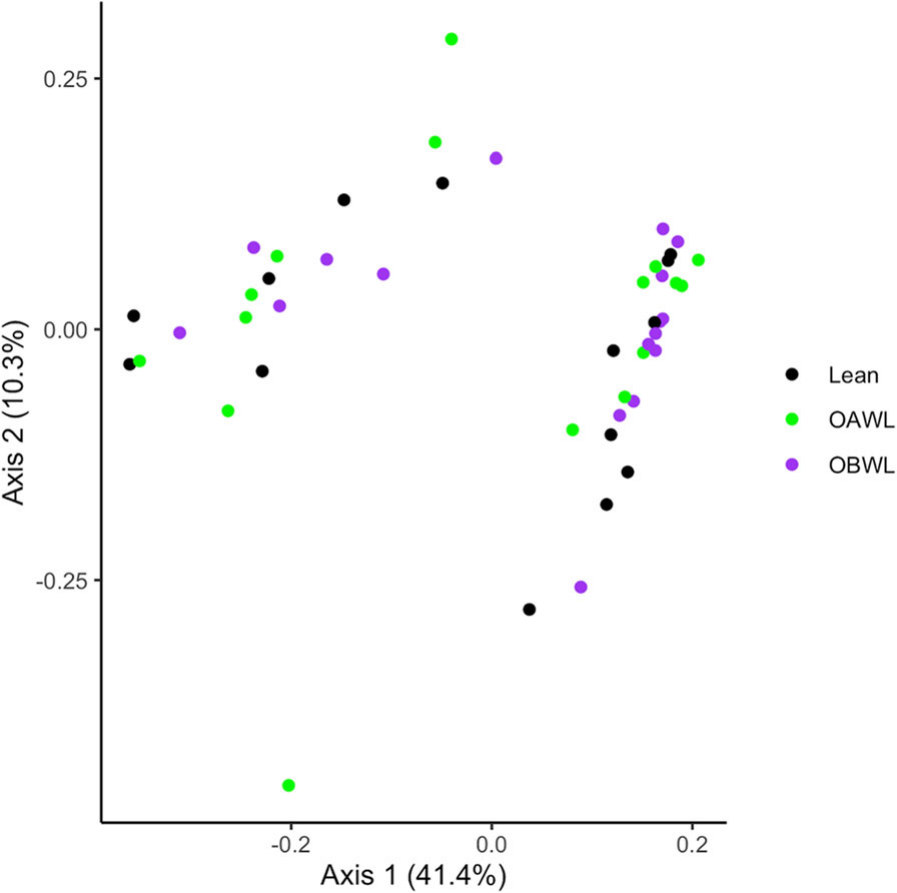
Three dimensional principal coordinate analysis of population membership of the fecal microbiota of healthy lean cats (LEAN, *n* = 14) and obese cats (OBWL, *n* = 17) following a 4-week adaptation period with a veterinary therapeutic food intended for weight loss and adult maintenance, and obese cats after a 10-week weight loss period on the same food (OAWL, *n* = 16). Each group is represented with a different colour (see legend)

No significant clustering per group was evident in a dendogram of the Classical Jaccard index representing the community membership of the fecal microbiota, and there was a strong intra-cat relationship (Fig. 4). Principal coordinate analyses also demonstrated no apparent clustering based on community membership (Fig. 5) or structure between groups (analysis of molecular variance (AMOVA) *P* > 0.05 and homogeneity of molecular variance (HOMOVA) *P* > 0.05 for all comparisons).

#### Linear discriminatory analysis effective size (LEfSe)

LEfSe analysis failed to identify enriched taxa between the OBWL and OAWL time-points. However, a comparison of the LEAN to the OBWL group revealed 13 OTUs that were differentially enriched in the OBWL group (Table 4), six of which are members of the phylum Firmicutes, two that belong to the phylum Proteobacteria and two to the phylum Actinobacteria. One OTU of the phylum Tenericutes was found differentially enriched in the LEAN group. When comparing LEAN to the OAWL cats, the only difference was enrichment of *Pseudomonas* (phylum Proteobacteria).

**Table 4:** Significantly enriched operational taxonomic units (OTUs) of the fecal microbiota of lean cats and obese cats before and after weight loss

Comparison	Significantly enriched OTUs		
	LEAN	OBWL	OAWL
LEAN vs. OBWL	Tenericutes *Anaeroplasma*	Firmicutes Unclassified Clostridiales (5 OTUs) *Planococcaceae_incertae_sedis* TM7 *TM7_genus_incertae_Sedis* Actinobacteria *Arthrobacter* *Nocardioides* Bacteroidetes Unclassified Prevotellaceae Verrucomicrobia Unclassified Verrucomicrobiaceae Proteobacteria *Oligella* Unclassified Proteobacteria	
LEAN vs. OAWL			Proteobacteria *Pseudomonas*

*OTUs* operational taxonomic units, *LEAN* lean cats, *OBWL* obese cats before weight loss, *OAWL* obese cats after weight loss

The OTUs represented here are from the fecal microbiota of healthy lean cats (LEAN, *n* = 14) and obese cats (OBWL, *n* = 17) following a 4-week adaptation period with a veterinary therapeutic food intended for weight loss and adult maintenance, and obese cats after a 10-week weight loss period on the same food (OAWL, *n* = 16). The presented OTUs have a linear discriminant analysis score > 2, and are organized in a descending manner

## Discussion

Based on human and rodent studies, it was expected that there would be differences of the fecal microbiota between lean and obese cats and that these microbial differences would revert with weight loss in obese cats. However, the bacterial fecal microbiota was only minimally affected in the current study.

One study reported on a 50% decrease in the relative abundance of Bacteroidetes in genetically-modified obese mice compared to lean mice that were fed the same high-polysaccharide diet. This change was also associated with a corresponding increase in Firmicutes and reduced diversity [18]. Similar changes were subsequently identified in obese adult humans [19]. In addition, weight loss, induced by using fat and carbohydrate-restricted diets, was found to restore the original ratio between Firmicutes to Bacteroidetes with the restoration being correlated to the percentage of weight loss [19]. More recent findings indicate that diet composition rather than caloric intake, had the greatest effect on the fecal microbiota of mice, when comparing lean mice to obese mice after weight loss (i.e., two groups of mice with similar weight and body composition) [34]. These findings corroborated other studies that showed that dietary effects (especially fat content) and initial body composition during weight reduction of obese mice and humans were the main contributors to a significant change in the fecal microbiota [35–37]. In contrast to those previous findings, such a broad effect was not noted in the current feline study as the relative abundance of taxa and biodiversity indices were not different between lean and obese cats, and in obese cats before and after weight loss. This could relate to various factors, such as differences between cats and other species, environmental influence, the pathophysiology of obesity in cats, the degree and duration of the initial obese status, the nature of the weight loss intervention, degree of cooperation of owners and the duration of the study period. Although in the current study, the weight loss period lasted only 10 weeks and the final fecal sample was taken when weight loss was still occurring, the food used was the same for all groups/periods, excluding diet as a confounder.

Despite the lack of broad taxonomic changes through comparison of relative abundances, differences in the microbiota were identified when comparing lean to obese cats before weight loss. Six of the 13 OTUs that were significantly enriched in obese cats before weight loss belong to Firmicutes, a finding which is consistent with the over-representation of certain Firmicutes members in obese subjects in other species [18]. In humans such an obesity-associated increase in Firmicutes was thought to be related to an increased ability of energy harvest and storage [18]. Additional research in genetically-related obesity in humans, as well as in mice fed a high-fat diet, was not able to correlate microbiota composition and membership changes with energy-harvest markers (i.e., short chain fatty acids (SCFA) and energy content in feces). Therefore, it was suggested that the changes observed indicated a potential microbial adaptation to diet or obesity over time, and hence, a more complex, and not completely understood, interaction between the microbiota and energy harvesting [38]. In a recent feline study, the influence of neutering, body composition, and 6 weeks of energy restriction on fecal microbiota of cats, was assessed. All cats were adapted to the study diet for 8 weeks before samples were obtained. No major differences were observed between groups in regards to bacterial composition and structure, however, a significant small difference in the abundance of Firmicutes between the lean neutered group to the obese group before energy restriction was observed, and unlike the common literature and the current study, they observed increased Firmicutes abundance in the lean neutered cats [25]. The authors of the mentioned study interpreted these finding as supportive to the idea that alterations in abundance at lower taxonomic level, such as genus, may have more impact and health effects compared with alterations in abundance at higher taxonomic level (e.g. phylum) [25]. The authors of the current study concur with this notion, despite the difference in findings.

In the current study, Planococcaceae *incertae sedis,* a taxa that belongs to the Planococcaceae family, was found to be enriched in obese cats before weight loss compared to lean cats. Two unclassified members from this family were also found to be enriched in pigs that had a higher residual feed intake, meaning, they were more efficient in energy harvesting [38]. In obese twins, a lower proportion of Bacteroidetes versus Actinobacteria was found, although the abundance of Firmicutes was not different. The same study identified obesity-related genes from the microbiome and found that 75% of the enriched obesity-related genes in obese twins originated from Actinobacteria, whereas the residual 25% originated from Firmicutes. Many of these genes’ functions are related to carbohydrate, amino-acid or lipid metabolism [37]. In the current study, Firmicutes, as well as Actinobacteria, were enriched in obese cats before weight loss compared to lean cats. In humans, obesity-specific Prevotellaceae, which belongs to the phylum Bacteroidetes, as well as some families that belong to the phylum Proteobacteria were enriched in morbidly obese individuals compared to individuals who went through gastric bypass or had a normal weight. Enrichment of Prevotellaceae and Proteobacteria was also demonstrated in obese cats before weight loss compared to lean cats enrolled in the current study. The Prevotellaceae family has members that facilitate protein and carbohydrate fermentation, as well as acetate, H_2_ and folate producers – hence, may have implications on energy metabolism and intestinal health [39, 40]. At last, in contrast to the current study, Verrucomicrobia was more abundant in individuals with a normal body weight or in individuals who had gastric bypass surgery, rather than in obese people [40]. Nonetheless, further understanding of the interaction between obesity and microbial features in the different species requires metagenomics research to further explore the obesity-related metabolic pathways and functional potential of the microbiome.

In addition, interesting complementary findings were observed when examining the effect of feline obesity and weight loss on serum cobalamin and folate concentrations. Serum cobalamin concentrations were lower in obese cats both before and after weight loss when compared to lean cats. Also, serum folate concentrations were higher in obese cats before weight loss compared to after weight loss. Both, serum cobalamin and folate concentrations, were within the laboratory’s reference interval in all cats, although for folate, concentrations were on the high end of the reference interval for obese cats after weight loss. A correlation between BW and serum cobalamin concentrations could not be demonstrated in a previous feline study [41], while in humans obesity is associated with a risk for cobalamin deficiency [42]. One of the suggested reasons for obesity-associated cobalamin deficiency is the diet consumed by obese humans, which is commonly richer in carbohydrates and fat, and lower in animal-derived protein, i.e., a diet lower in cobalamin [43]. Still, low cobalamin intake was not expected in the current study. First, as strict carnivores, cats consume a diet high in animal-derived protein and therefore also in cobalamin. Second, the cats were fed a complete and balanced diet formulated for adult maintenance and intended for weight loss, meaning that protein and micronutrient content were enhanced to compensate for reduced energy intake. Hence, it is possible that no true cobalamin deficiency was observed due to the consumption of a cobalamin-enriched diet.

Serum cobalamin and folate concentrations are indicators for gastrointestinal disease in humans [44]. In cats and dogs, a similar association has been suggested due to the positive therapeutic outcome of intestinal disease when cobalamin concentrations are replenished [28, 29]. In general, both cobalamin and folate are affected by intestinal health and more specifically intestinal absorptive capacity of various segments of the small intestine (i.e., folate in the proximal small intestine and cobalamin in the ileum) [26, 28, 29]. Considering an association between serum cobalamin and folate and intestinal health and absorption, the current findings, whilst still being within the normal reference interval, may suggest better intestinal properties in lean compared to obese cats and improvement of intestinal health with weight loss.

The presence of enriched taxa in obese cats prior to weight loss, compared to lean cats, as well as the failure to identify such differences in enriched taxa between obese cats before and after weight loss, may strengthen the findings that body composition and % weight loss, while excluding diet as a confounder, are the main drivers of changes in the fecal microbiota [34–37]. Although BW and BCS significantly decreased with energy restriction, the weight loss observed may not have been sufficient to cause significant changes in the biodiversity indices, relative abundance or LEfSe analysis in obese cat over the course of the weight loss plan. Still, fewer differences in enriched taxa were observed when comparing obese cats after weight loss to the lean cats. This might mean that with weight loss, the microbial population membership and composition are changing slowly, yet a 10-week weight loss period might not be long enough to completely revert the obesity-related differences in enriched taxa to a lean body condition.

Challenges occurred with fecal collection during the current study. The owners were advised to maintain the fecal samples at 4 °C until transfer to the clinic, yet, some owners kept them at ambient temperature. In a study by Weese and Jalali (2014), no changes in microbial membership and composition were observed during 7 day storage at 4 °C [45]. Likewise, a recent study investigated the effects of storage of fecal samples at ambient temperature on the feline fecal microbiota and confirmed the validity of using feline fecal samples that were kept at an ambient temperature for up to 4 days for microbiota-related analyses [46]. Therefore, despite the mentioned challenges, the authors did not expect fecal samples storage conditions to affect the results of the microbial-related analyses.

In the current study, aside to the relatively short weight loss period that was not sufficient for obese cats to reach their ideal body weight, the sample size was relatively small for all groups, and can be considered a limitation. However, since a similar study had not previously been conducted in client-owned cats, the samples size was aligned with other feline studies, investigating the effects of GI inflammatory conditions on the microbial population [47, 48]. Nevertheless, it is possible that with a larger sample size, and a longer period of caloric restriction would promote greater weight loss as well as more distinct and consistent changes in the fecal microbiota than were identified in the current study here.

## Conclusions

Enriched taxa in LEfSe analysis were observed especially when comparing obese cats before and after weight loss. Nearly half of the enriched taxa in the obese group belonged to Firmicutes, which concurs with previous reports in humans and mice. This may be related to better energy-harvesting abilities of the host. However, a metagenomic approach is warranted to explore the functional potential of the feline obese fecal microbiome. The current study also demonstrated minimal effects of a 10-week standardized and successful weight loss plan on microbial biodiversity, while excluding diet as a confounder. This may imply that sudden short-term energy restriction is not enough to revert microbial changes in the intestines.

Moreover, assessment of serum cobalamin and folate, although all concentrations were within the reference interval, potentially suggest improved intestinal health in lean compared to obese cats, which could be achieved with weight loss in obese cats. Nevertheless, more research is warranted allowing for a longer weight loss period and a larger sample size.

## Methods

### Experimental design

Fourteen lean (BCS 4–5/9, 10 males and 4 females) and 17 obese (BCS ≥ 8/9, 11 males and 6 females) cats were enrolled into the study [49]. All cats were client-owned animals from the Guelph, Ontario region, who live indoors, were neutered and between 2 and 9 years of age. All cats were assessed to be healthy, apart from obesity, based on physical examination and medical history provided by the cat’s owner, as well as complete blood count (CBC) and serum biochemistry profile. There was no history of antimicrobial or anti-inflammatory medications administered in the 90 day period before commencing study enrolment. The study took place between May 2015 and December 2016.

### Adaptation period

At the time of enrolment, BW, BCS and muscle condition score (MCS) [49, 50] were documented. Next, all cats underwent a one-week transition period to a veterinary therapeutic food (Hill’s Prescription Diets Metabolic Feline dry, Hill’s Pet Nutrition, Topeka, Kansas, USA) intended for weight loss and adult maintenance (Table 5), followed by a 4-week adaptation period during which all cats received the study food for 100% of their daily ration. Individual maintenance energy requirements (ER) for both lean cats (LEAN) and OBWL were calculated in accordance with the National Research Council (NRC) (LEAN: 100 Kcal/kg^0.67^; OBWL: 130 Kcal/kg^0.4^) based on ideal body weights [51]. The owners recorded the daily food intake of each cat. Three weeks following study enrolment, body weight was assessed, and the amount of food offered was adjusted, aiming to maintain a stable body weight. At the end of the adaptation period (week 5) BW, BCS and MCS were recorded. Body condition score was once again evaluated using a 9-point scale, previously validated for cats [49]. Muscle condition score was assessed using a 4-point scale, which was previously described and validated for cats [52]. Also, BMI and girth were assessed in all cats at that time. Body mass index was calculated according to a previous study [53] and girth was measured right behind the last rib [54]. The same investigator (MT) conducted all measurements throughout the study to reduce variability. A blood sample was drawn from the jugular or cephalic vein for analysis of serum cobalamin and folate concentration. Fecal samples were collected by the owners and were obtained by the study personnel within 24 h after defecation and frozen at − 80 °C until further analysis. The lean cats completed the study at week 5, while the obese cats continued with a 10-week weight loss plan.

**Table 5:** Proximate and total dietary fibre analyses of the veterinary therapeutic food fed in this study^a^

	Units	Content^b^
Moisture	g/100 g	5.5
CF (by acid hydrolysis) (DM)	g/100 g	13.0
CP (DM)	g/100 g	38.6
NFE (DM)^c^	g/100 g	36.3
Cf (Cf) (DM)	g/100 g	6.3
Total dietary fibre (DM)	g/100 g	18.5
CA (DM)	g/100 g	5.8
Energy density (DM)^d^	kcal/100 g	372.7

*CF* crude fat, *CP* crude protein, *NFE* nitrogen-free extract, *Cf* crude fibre, *DM* dry matter, *CA* crude ash

The food was fed as the only food source to lean cats (LEAN, *n* = 14) for adult maintenance for 4 weeks and to obese cats for adult maintenance for 4 weeks (OBWL, *n* = 17), followed by a 10-week weight loss period (OAWL, *n* = 16)

^a^Hill’s Prescription Diets Metabolic Feline (dry), which contained chicken by-product meal, brewers rice, corn, gluten meal, powdered cellulose, dried tomato, pomace, flaxseed, dried beet pulp, chicken liver flavor, coconut oil, pork fat, lactic acid, potassium chloride, calcium sulfate, L-lysine, choline chloride, carrots, DL-methionine, vitamins (vitamin E supplement, L-ascorbyl-2-polyphosphate (source of vitamin C), niacin supplement, thiamine mononitrate, calcium pantothenate, pyridoxine hydrochloride, vitamin A supplement, riboflavin supplement, biotin, vitamin B12 supplement, folic acid, vitamin D3 supplement), taurine, L-carnitine, minerals (manganese sulfate, ferrous sulfate, zinc oxide, copper sulfate, calcium iodate, sodium selenite), mixed tocopherols for freshness, natural flavors, β-carotene

^b^Nutrient content refers to an average of two consecutive laboratory analyses from the same bag, which were performed by Maxxam Analytics International Corporation, Mississauga, Ontario, Canada;

^c^Calculated using the equation: NFE (g/100 g) = 100 – (CP + CF + Cf + CA) [51]

^d^Calculated using the equation: Energy density (kcal/100 g) = (CF × 8.5) + (CP × 3.5) + (NFE × 3.5) [51]

### Weight loss period

At week 5, the individual ER for weight loss were calculated for the obese cats in accordance with NRC recommendations (0.6 × 130 Kcal/kg^0.4^), based on ideal body weight. The weight loss period continued for 10 weeks. During this period, food intake was recorded daily by the owners and BW, BCS and MCS were assessed every other week to monitor for effective and safe weight loss (with a target BW loss of 0.5 to 2% of the initial BW per week [9, 55]), as well as to monitor maintenance of lean body mass. If the weekly weight loss rate was below 0.5% or exceeded 2% of the initial BW, individual ER adjustments were made – lowering or increasing the ER initially by 5%, respectively [12]. After 10 weeks, assessment of BMI and girth were repeated. At that time, additional blood samples were collected, food logs obtained from the owners. Fecal samples were also collected by the owners for the obese cats after weight loss (OAWL) and fecal samples were stored as described above.

### Laboratory analyses of blood samples

Blood was collected at the end of the adaptation period (LEAN and OBWL) and at the end of the weight loss period (OAWL) for analyses of serum concentrations of cobalamin and folate was refrigerated at 4 °C for 2 h and then centrifuged at 3000 rpm for 10 min (Sorvall Legend RT Centrifuge. Fisher Scientific, Ottawa, Canda). The serum retrieved was aliquoted and frozen at − 80 °C. Frozen serum samples were batched from both the lean and obese groups, and were analyzed together. Serum concentrations of cobalamin and folate were analyzed at the Gastrointestinal Laboratory, Texas A&M University, using a chemiluminescent enzyme immunoassay (Immulite 2000 Immunoassay System. Siemens Healthcare Systems GmbH, Erlangen, Germany) involving an automated alkaline denaturation procedure.

### Sample preparation and DNA extraction

All fecal samples, collected at the end of the adaptation period (LEAN and OBWL) and at the end of the weight loss period (OAWL), were analysed simultaneously. Whole fecal samples were thawed overnight in their original container in a refrigerator (+ 4 °C), manually homogenized in a biosafety cabinet (Class II, Type A2 Biosafety Cabinet, Thermo Fisher Scientific, Waltham, Massachusetts, USA) and aliquoted into 200 mg samples.

DNA extraction was conducted using a commercial stool extraction kit (E.Z.N.A. Stool DNA Kit, Omega Bio-Tek Inc., Doraville, Georgia, USA) in accordance with the manufacturer’s instructions. Extracted DNA samples were stored at − 80 °C until further analysis.

### Polymerase chain reaction (PCR)

A spectrophotometer (NanaDrop 1000 Spectrophotometer, Nano Drop Technologies Inc. (Thermo Fisher Scientific), Waltham, Massachusetts, USA) was used to assess the quantity of extracted DNA. All DNA samples were diluted (if needed) to a range of 30 to 100 ng/ml. The V4 region of the 16S rRNA gene was amplified using a PCR with the forward primer: S-D-Bact-0564-a-S-15 (5′-AYTGGGYDTAAAGNG-3′), reverse: S-D-Bact-0785-b-A-18 (5′-TACNVGGGTATCTAATCC-3′) [56], KAPA HiFi ReadyMix (Kapa Biosystems, Wilmington, Massachusetts, USA), and PCR grade water. The PCR products were purified with Agencourt AMPure XP (Beckman Coulter Inc., Mississauga, ON, Canada). In order to prepare the PCR products for Illumina MiSEq (Illumina, San Diego, California, USA) sequencing, the purified PCR products were amplified by PCR with Illumina adapters (Mastercycler Pro, Eppendorf Canada Ltd., Mississauga, Ontario, Canada) and then once again purified. Prior to Illumina sequencing, the finalized PCR products were evaluated using gel electrophoresis and DNA was measured using spectrophotometry.

### DNA sequencing

Bridge amplification was performed with an Illumina MiSeq system (Illumina, San Diego, California, USA), using terminator nucleotides that were incorporated into the amplified PCR products with the removal of the terminator group [57].

### Bioinformatics and statistical analyses

Following DNA sequencing of fecal samples, Mothur v1.36.1 was used for sequence processing [58, 59]. Assembly of paired end reads was performed using the make.contigs command. This command extracts the sequence and creates its reverse compliment and joins the reads into contigs. Next, filtration was conducted using several screen.seqs commands to remove sequences greater than 250 bp in length and those with any ambiguous base calls or runs of homopolymers greater than 8 bp. Alignment of sequences to the Silva v128 16S rRNA reference database [60] was implemented, with the removal of sequences that did not align with the correct region. Uchime was conducted using the same Silva reference database to identify chimeras [61], which were then removed. Archaea were removed as well using the remove. Lineage command. A closed OTU picking approach was then used. Ribosomal Database Project (RDP) classifier (v14) was used for taxonomic assignment of sequences [62], and the reference database used was v11.4. In order to standardize sequence numbers used for analysis, subsampling was completed based on the smallest number of sequences from a sample [63].

Further statistical analyses were performed using JMP 13.0 (SAS Campus Drive, Cary, North Carolina, USA). Normality of data distribution was assessed using the Shapiro-Wilk test. Evenness, diversity and richness were calculated using Shannon diversity [64], Simpson diversity [65] and Chao [66] respectively, and a nonparametric multiple comparison test (Wilcoxon Rank Sum) was used to compare between the groups and time points (LEAN, OBWL and OAWL). Relative abundances were calculated for the different taxonomic levels, for each group. The relative abundance threshold for phyla was above 1% and above 0.1% for the rest of the taxa levels. Differences were evaluated using nonparametric multiple comparison test (Wilcoxon Rank Sum for comparisons between groups: LEAN to OBWL and LEAN to OAWL; Wilcoxon Signed-Rank for the comparison between time points: OBWL to OAWL), with *p*-values adjusted using the Benjamini-Hochberg correction (SAS Campus Drive, Cary, North Carolina, USA; R. Core Team, 2013, R Foundation for Statistical Computing, Vienna, Austria) to control for false discovery [67]. Relative abundances are presented as median with range (minimum to maximum). The classical Jaccard index [68] and Yue & Clayton index of dissimilarity [69] (beta-diversity indexes) were calculated to examine community membership and population structure, respectively. To reflect the differences in membership and structure between the groups, dendrograms were generated, and significance of clustering according to group was determined using parsimony and unweighted unifrac tests [70]. Beta-diversity indices were also visualized using principal coordinate analyses (PCoA), with further comparison of groups by AMOVA and HOMOVA. In order to identify difference in taxa between all groups, linear discriminatory analysis (LDA) effective size (LefSe) [71] was conducted.

Statistical analyses for body weight, body composition measurements and serum cobalamin and folate concentrations were performed using SAS v9.3 (SAS Campus Drive, Cary, North Carolina, USA). Normality of the data was assessed using the Shapiro-Wilk test. Data that did not distribute normally were logged, and if the distribution was still not normal, then non-parametric tests were performed. Differences in BW, BCS, BMI and girth measurements, as well as serum cobalamin and folate concentrations between LEAN to OBWL and OAWL groups were compared using a student T-test/paired T-test or the corresponding Wilcoxon-Mann-Whitney/Wilcoxon Signed-Rank, depending on normality and on whether the samples were paired. Significance was set at *P* < 0.05 for all comparisons. Normally distributed data are expressed as mean SD (BW, BMI, girth measurements) or as mean of the back transformed values (lower limit (LL)-upper limit (UL)) (folate). Data that did not have a normal distribution are presented as median with range (minimum to maximum) (cobalamin, BCS).

## Data Availability

The dataset generated and/or analysed during the current project is available at the Scholars Portal Dataverse server (https://dataverse.scholarsportal.info/dataset.xhtml?persistentId=doi:10.5683/SP/9VAK4K).

## Abbreviations

GI: Gastrointestinal
PCR: Polymerase chain reaction
LPS: Lipopolysaccharide
OBWL: Obese cats before weight loss
OAWL: Obese cats after weight loss
BW: Body weight
SD: Standard deviation
BCS: Body condition score
BMI: Body mass index
OUT: Operational taxonomic unit
LEfSe: Linear discriminatory analysis effective size
SCFA: Short chain fatty acids
CBC: Complete blood count
MCS: Muscle condition score
ER: Energy requirements
RDP: Ribosomal Database Project
PCoA: Principal coordinate analyses
HOMOVA: Homogeneity of molecular variance
AMOVA: Analysis of molecular variance
LDA: Linear discriminatory analysis
LL: Lower limit
UL: Upper limit
